# Uncovering fertility potential: Clinical and histopathological insights from testicular biopsies in azoospermic men in a large Indonesian cohort

**DOI:** 10.12688/f1000research.166812.1

**Published:** 2026-01-07

**Authors:** Dyandra Parikesit, Armand Achmadsyah, Favian Ariiq Rahmat, Retta Catherina Sihotang, Heri Wibowo, Aria Kekalih, Luluk Yunaini, Asmarinah, Eric Chung, Ponco Birowo

**Affiliations:** 1 Department of Urology, Universitas Indonesia Hospital, Depok, West Java, 16424, Indonesia; 2 Doctoral Program in Medical Sciences Faculty of Medicine, Universitas Indonesia, Central Jakarta, Jakarta, 10430, Indonesia; 3 Faculty of Medicine, Universitas Indonesia, Central Jakarta, Jakarta, 10430, Indonesia; 4 Department of Urology, Cipto Mangunkusumo Hospital, Central Jakarta, Jakarta, 10430, Indonesia; 5 Metabolic Disorder, Cardiovascular, and Aging Research Center, Indonesia Medical Education & Research Institute (IMERI), Faculty of Medicine, Universitas Indonesia, Central Jakarta, Jakarta, 10430, Indonesia; 6 Community Medicine Department, Faculty of Medicine, Universitas Indonesia, Central Jakarta, Jakarta, 10430, Indonesia; 7 Department of Medical Biology, Faculty of Medicine, Universitas Indonesia, Central Jakarta, Jakarta, 10430, Indonesia; 8 Department of Urology, Princess Alexandra Hospital, University of Queensland, Brisbane, QLD, 4102, Australia; 9 Indonesian Reproductive Science Institute, Bunda Hospital, Central Jakarta, Jakarta, 10350, Indonesia

**Keywords:** Azoospermia, infertility, male, histology, sperm retrieval

## Abstract

**Objective:**

This study aims to evaluate spermatogenic failure in azoospermic men by characterizing patterns in Modified Johnsen Scores from testicular biopsies, examining their correlation with clinical and hormonal parameters, and determining the probability of live sperm retrieval.

**Novelty:**

As the largest dataset on azoospermia in Indonesia, this study provides a comprehensive histopathological and hormonal profile of azoospermic patients, while also emphasizing the diagnostic value of bilateral testicular biopsies—an area often underexplored in Southeast Asian populations.

**Methods:**

A retrospective analysis was conducted on azoospermic patients who underwent bilateral testicular biopsies between April 2011 and July 2024. Testicular tissue samples were assessed using the Modified Johnsen scoring system (range: 1 to 10), and classified histopathologically from tubular fibrosis to complete spermatogenesis. Clinical parameters including age, body mass index, sperm retrieval outcomes, and serum levels of follicle-stimulating hormone, luteinizing hormone, and testosterone were collected and analyzed. Statistical associations between these parameters and histopathological subtypes were determined using significance testing.

**Results:**

A total of 409 testicular biopsies were evaluated. The most frequent Modified Johnsen Scores were 7 (20%) and 5 (16.6%). Histopathological analysis showed that 40.3% of cases exhibited spermatogenic arrest, while only 7% demonstrated normal spermatogenesis. Discordant histopathological patterns between the two testes were observed in 18.5% of patients. Follicle-stimulating hormone and luteinizing hormone levels showed statistically significant associations with histopathological subtypes (p < 0.001). Age, body mass index, and testosterone levels did not correlate significantly.

**Conclusion:**

Spermatogenic arrest is the predominant abnormality in azoospermic men, with hormonal variations—particularly in follicle-stimulating hormone and luteinizing hormone—strongly associated with testicular pathology. The presence of discordant histology between testes underscores the importance of bilateral biopsies. Higher Modified Johnsen Scores were positively correlated with the likelihood of successful sperm retrieval.

## Introduction

Infertility affects a significant proportion of couples worldwide and has been reported in around 17.5% of adults worldwide, according to the World Health Organization (WHO). ^1^ Among male infertility cases, 10–15% of males reported having azoospermia—a condition characterized by the absence of sperm in ejaculate—which represents a severe manifestation that challenges fertility interventions. ^2^ Supporting this, a study conducted in Jakarta, Indonesia involving 1,062 male infertility patients revealed that only 5.13% had normal semen parameters, with the majority of abnormalities attributed to azoospermia. Beyond its clinical significance, male infertility carries profound psychosocial, economic, and reproductive consequences. Affected individuals often experience psychological distress, social stigma, and a diminished sense of self-worth. Economically, infertility imposes substantial costs, particularly in low- and middle-income countries where access to assisted reproductive technologies (ART) remains limited. ^3^ Understanding the underlying causes and identifying fertility potential in azoospermic men have led to increasing reliance on testicular biopsy as a diagnostic and prognostic tool.

The diagnostic evaluation of male infertility generally begins with hormonal profiling, semen analysis, and scrotal ultrasonography. Hormonal tests—particularly levels of follicle-stimulating hormone (FSH), luteinizing hormone (LH), and testosterone—offer insights into the endocrine regulation of spermatogenesis. The histopathological evaluation of testicular biopsies offers crucial insights into spermatogenic function. Research has revealed that despite severe testicular dysfunction, isolated spermatogenic foci are often present. This allows assisted reproductive technologies (ART) such as testicular sperm extraction (TESE) or micro-TESE (TESE using the aid of a surgical microscope) combined with intracytoplasmic sperm injection (ICSI) to achieve successful fertilization and pregnancy outcomes. ^4^ Moreover, the ability to differentiate obstructive from nonobstructive azoospermia through endocrine and histological assessments has refined patient counselling and treatment approaches. ^5^

The success rate of the micro-TESE procedure is associated with the characteristics of the treated population, primarily age and histological patterns in the testes. ^6^ A comprehensive histopathological report from a diagnostic testicular biopsy is essential for the prognosis of the micro-TESE procedure. ^7^ The predominant morphological patterns observed in the testicular biopsies of NOA patients after unsuccessful sperm retrieval during a micro-TESE procedure include hypospermatogenesis (HS), Spermatogenic Arrest (SA), Sertoli Cells Only Phenotype (SCOP), and Tubular Fibrosis (TF). These patterns represent varying degrees of spermatogenic failure, each with distinct implications for fertility outcomes. For instance, patients with hypospermatogenesis typically have the highest chance of sperm retrieval, while those with tubular fibrosis have the lowest. Moreover, these histopathological findings often correlate with hormonal profiles. Elevated follicle-stimulating hormone (FSH) and luteinizing hormone (LH) levels are commonly associated with severe germ cell depletion seen in SCOP and TF, whereas more favorable patterns like HS may present with relatively normal hormonal levels. However, such correlations are not absolute. Hormonal markers and testicular size alone often fail to accurately predict the presence of focal spermatogenesis. Consequently, histopathological analysis is essential for appropriately selecting patients for the “second look” micro-TESE procedure, enhancing the likelihood of successful infertility treatment.

In Indonesia, a country with unique demographic and healthcare challenges, investigating testicular biopsy outcomes in azoospermic men provides valuable insights into the interplay between clinical, endocrinological, and histopathological variables. Despite global advances in male infertility research, Southeast Asia remains underrepresented in the literature, with a notable lack of large-scale studies exploring testicular histopathology among azoospermic populations. The prevalence among Indonesia or other Southeast Asian countries remain unavailable. This study aims to uncover fertility potential within a large cohort of Indonesian men by analyzing testicular biopsy results, exploring correlations with endocrine parameters, and evaluating the utility of these findings for improving ART outcomes. Given Indonesia’s vast population, genetic diversity, and regional disparities in healthcare access, population-specific data are urgently needed to develop locally relevant diagnostic algorithms and fertility treatment strategies.

## Methods

### Study design and setting

This cross-sectional study included testicular biopsies from patients diagnosed with non-obstructive azoospermia (NOA) who underwent testicular sperm extraction (TESE) or micro-TESE. The procedures were performed at two tertiary care centers in Jakarta, Indonesia: Cipto Mangunkusumo National Referral Hospital and Bunda General Hospital, spanning from January 2009 to March 2024.

### Ethics considerations

The study design was approved by the Health Research Ethics Committee – Faculty of Medicine Universitas Indonesia and Cipto Mangunkusumo Hospital (HREC-FMUI/CMH), which waived the requirement for obtaining informed consent, as this was a retrospective study utilizing fully anonymized clinical and histopathological data. The approval was granted under the ethical No. KET-1719/UN2.F1/ETIK/PPM.00.02/2024; Protocol ID: 24-11-1740.

### Sample size and sampling method

This study employed a retrospective cross-sectional design using total sampling of azoospermic patients who underwent testicular biopsy at RSUPN Cipto Mangunkusumo between January 2010 and November 2024. Sample size was calculated using a correlation formula with a significance level of 95% (Zα = 1.96), power of 80% (Zβ = 0.84), and assumed correlation coefficient (r) of 0.5. Based on the formula:

$$n=\frac{{\left(Z_{\alpha /2}+Z_{b}\right)}^{2}\times {\left(1+r\right)}^{2}\;}{r^{2}}$$

The minimum required sample size was 71 patients. However, all eligible patients meeting the inclusion criteria were included to enhance the study’s power and representativeness.

### Eligibility criteria

*Inclusion criteria*

Azoospermic patients who underwent sperm retrieval procedures (TESE and micro-TESE) at Cipto Mangunkusumo National General Hospital (RSUPN Cipto Mangunkusumo) between 2010 and 2024. Patients were required to have complete data, including hormonal profiles (FSH, LH, testosterone), metabolic data (BMI, total cholesterol, HDL, LDL, triglycerides), ultrasonographic findings (presence of varicocele and the longest testicular axis), and testicular histopathological results.

*Exclusion criteria*

Patients with severe cardiac or pulmonary insufficiency, significant coagulation disorders, no prior history of sperm retrieval procedures, incomplete medical records, or those who refused consent for surgical procedures were excluded from the study.

### Data collection/study procedure

Patient demographics, including age and laboratory results, were extracted from medical records. All testicular specimens were processed using standard protocols: fixation in Bouin’s Fluid, staining with Hematoxylin and Eosin (HE), and histological assessment via light microscopy. Biopsies were classified into four histological patterns: hypospermatogenesis (HS), spermatogenic arrest (SA), Sertoli cell-only phenotype (SCOP), and tubular fibrosis (TF). ^6, 7^ Discordant biopsy was defined as differing histopathological patterns between the right and left testes.

**Figure 1.**
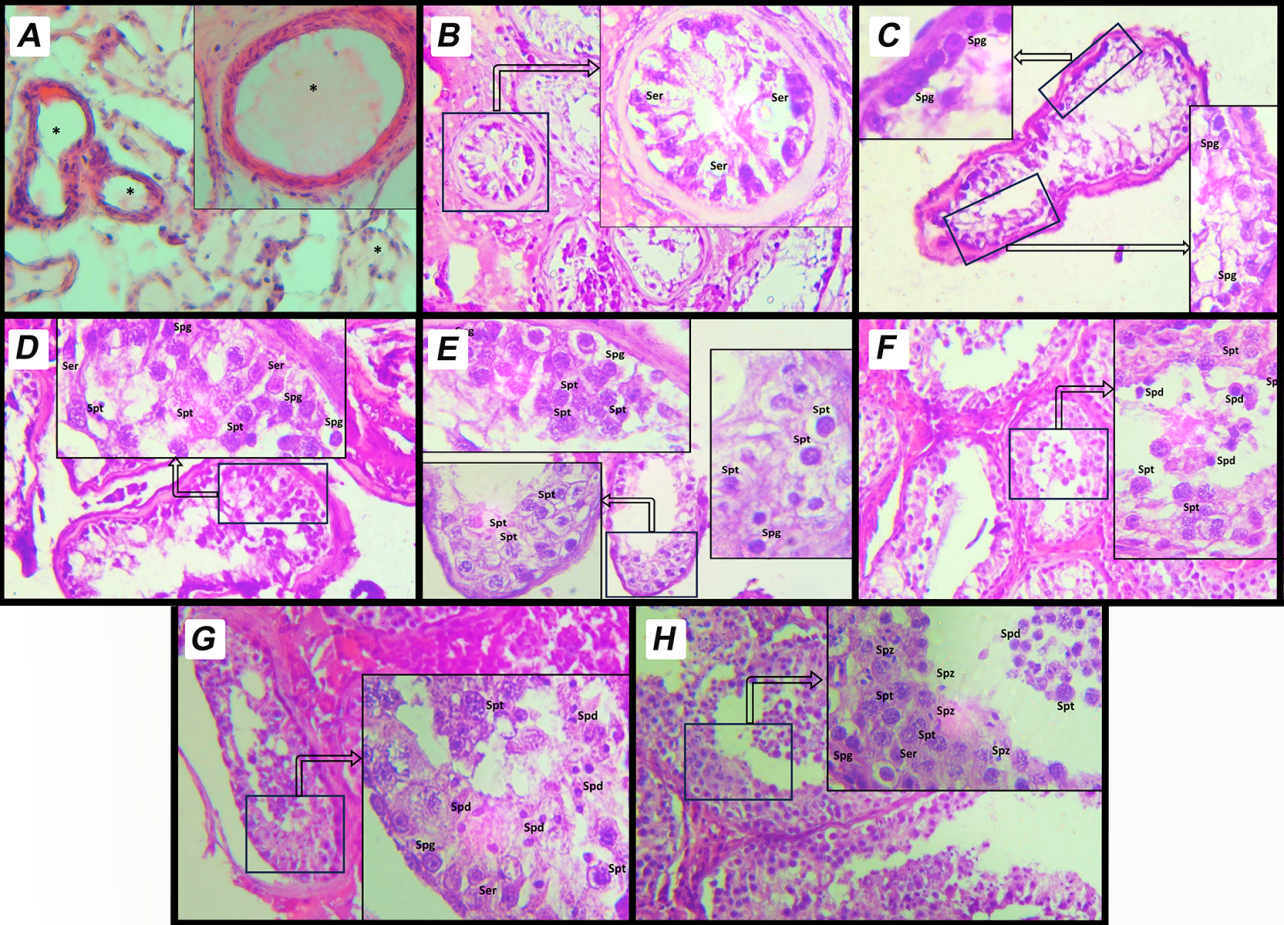
Histological representation of modified Johnsen scoring system on human testicular tissue. This image depicts a histological section of human testicular tissue evaluated using MJ scoring system. The scoring system assesses spermatogenic activity based on the arrangement and presence of different cell types within the seminiferous tubules. A. Score 9: Complete spermatogenesis with a dense layer of spermatozoa filling the tubule lumen. The tubule shows regular spermatogenic activity with all stages of germ cell development, including spermatogonia, spermatocytes, spermatids, and spermatozoa. B. Score 8: Complete spermatogenesis with spermatozoa present but in reduced density compared to score 9. C. Score 7: Presence of spermatids and earlier germ cell stages, but no spermatozoa observed in the tubule lumen. D. Score 6: Presence of spermatocytes and earlier germ cell stages, but no spermatids or spermatozoa. E. Score 5: Presence of spermatogonia only, without progression to more advanced stages of spermatogenesis. F. Score 4: Sertoli cells and very few spermatogonia, with no active spermatogenesis. G. Score 3: Only Sertoli cells are observed in the seminiferous tubules; all germ cells are absent. This is referred to as Sertoli-cell-only Phenotype. H. Score 2: Tubules contain minimal structures and show signs of degeneration, with severely atrophic features. Sertoli cells may be present, but germ cells are absent. I. Score 1: Complete fibrosis of the seminiferous tubules. Both germ cells and Sertoli cells are absent, indicating end-stage testicular damage. Sertoli Cells (Ser): Supporting somatic cells, identified by their elongated nuclei. Spermatogonia (Spg): Basally located germ cells involved in the initiation of spermatogenesis. Primary Spermatocytes (Spt): Larger cells undergoing meiotic division, positioned centrally within the tubule. Spermatids (Spd): Small, round cells indicative of advanced stages of spermatogenesis. Spermatozoa (Spz): Mature germ cells aligned along the lumen of the tubule. * Empty cell in seminiferous tubules.

Each testicular biopsy sample was evaluated using the Modified Johnsen (MJ) scoring system ( Table 1), a semi-quantitative scale ranging from 1 to 10 that reflects the degree of spermatogenic activity within seminiferous tubules. ^8^ A score of 10 indicates complete and normal spermatogenesis, while a score of 1 reflects complete absence of germ cells with tubular fibrosis. In cases where bilateral discordance was observed, the higher score (i.e., the side with more favorable histological features) was used for classification. For analytical purposes, scores were categorized as follows: normal spermatogenesis (MJ >8), hypospermatogenesis (HS) (MJ = 7), spermatogenic arrest (SA) (MJ 6–3), Sertoli cell-only phenotype (SCOP) (MJ = 2), and tubular fibrosis (TF) (MJ = 1). ^9, 10^ Representative histological images for each MJ score category were captured at 400× magnification and presented in Figure 1.

**Table 1.** Modified Johnsen score for assessment of spermatogenesis. ^1^

Score	Level of spermatogenesis
1	No seminiferous epithelium, prominent sclerosis
2	Only Sertoli cells present
3	Spermatogonia present without other element of spermatogenesis
4	Spermatogenesis arrested at the level of primary spermatocyte
5	Many spermatocytes, no sperm or spermatids
6	Only few early spermatids without late spermatids or spermatozoa
7	No late spermatids and sperm; many early spermatids
8	Reduced number of sperm with less than 5 spermatozoa per tubules and a few late spermatids
9	Incomplete spermatogenesis with many late spermatids
10	Full spermatogenesis

**Figure 2.**
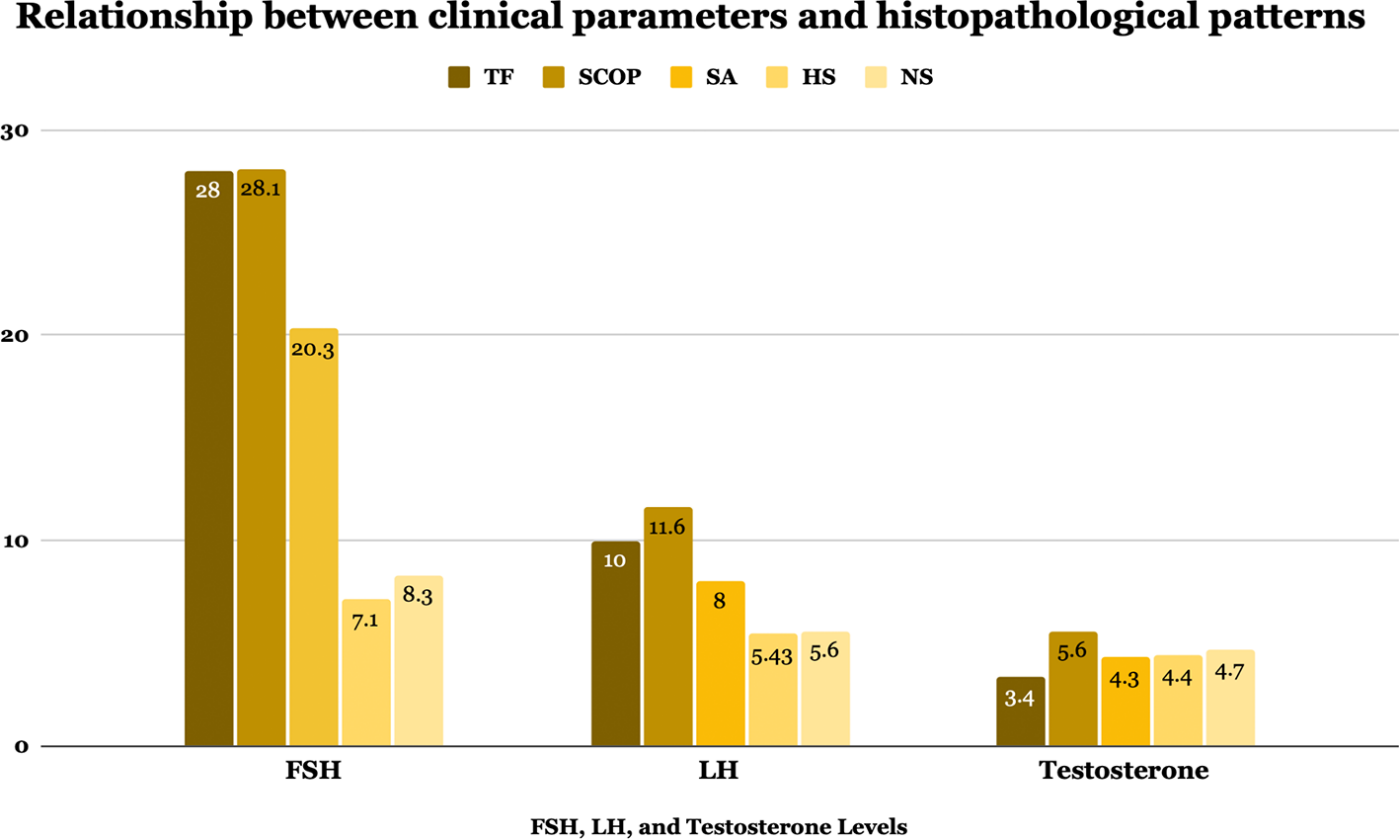
Hormonal profile levels in different histopathological groups.

### Study variables

Clinical and laboratory parameters evaluated included body mass index (BMI), Glycated hemoglobin (HbA1c), FSH, LH, and testosterone levels. BMI was calculated from recorded weight and height. HbA1c was considered normal below 5.7%. ^11^ Hormone reference ranges were: FSH (1.5–12.4 mIU/mL), LH (2–9 IU/L), and testosterone (2.49–8.36 ng/mL). These variables were analyzed for their potential correlation with histopathological findings.

*Blood collection and analysis*

Clinical and laboratory testing were done before the procedure and analyzed to evaluate its relationship with the histopathological results. Venous blood samples were obtained from all patients prior to the testicular biopsy procedure using standard sterile techniques. Blood was collected into serum separator tubes (SST) for hormonal assays (FSH, LH, testosterone) and EDTA tubes for HbA1c analysis. Serum samples were allowed to clot at room temperature, then centrifuged at 3000 rpm for 10 minutes. The serum was aliquoted into labeled cryotubes and stored at –20°C for short-term or –80°C for long-term preservation until analysis. EDTA samples for HbA1c were stored at 4°C and analyzed within 24 hours.

Hormonal assays were conducted in an accredited clinical laboratory using standard immunoassays. The normal reference ranges used were: FSH (1.5–12.4 mIU/mL), LH (2–9 IU/L), and testosterone (2.49–8.36 ng/mL). HbA1c values below 5.7% were considered normal. ^10^ All laboratory tests followed internal and external quality control standards to ensure reliability and reproducibility. BMI was calculated and recorded as continuous data. Metabolic markers including lipid profiles were also assessed to evaluate their association with histopathological outcomes.

### Statistical analysis

The data were statistically analyzed using IBM SPSS Statistics version 29.0.2.0. They were analyzed by type using the Chi-square test, multinomial logistic regression, and ANOVA test, as applicable. For multinomial outcomes, multinomial logistic regression was applied to identify associations between histopathological patterns and hormonal levels. A p-value of <0.05 was considered statistically significant. All tests were two-tailed.

## Results

A total of 409 testicular biopsy cases from bilateral testicular biopsies were performed. The mean age of patients was 36±6.7 years, ranging from 19 to 69 years. All of the included patients can be seen in Table 2. All the cases were categorized according to the histopathological categories mentioned in Table 3. Nearly half of the histological pattern was Spermatogenic Arrest (49.6%), followed by HS, seen in 20% of cases. However, according to individual MJ scores, the most common score is MJ score 2 (Only Sertoli cells present), followed by MJ score 5 (Many spermatocytes, no sperm or spermatids). There was a significant difference in sperm found during the procedure from TF (MJ 1) to SA (MJ 6–3) ( *p*=<0.001) but not HS (MJ 7) ( *p*=0.5), showing varying degrees of sperm retrieval probability, with odds ratios progressively decreasing as pathology severity increases. There were 76 patients (18.5%) who showed a discordant pattern.

**Table 2.** Characteristics of the included patients.

Parameters	Total N=409 (%)	TF N=30 (7.3%)	SCOP N=51 (12.5%)	SA N=203 (49.6%)	HS N=82 (20%)	NS N=10.5%
Age (years) (Mean±SD)	36.74±6.78	34.83±4.34	35.10±5.95	36.71±6.47	38.39±8.77	37.05±5.64
BMI (kg/m ^2^)	28.16±5.91	27.74±5.16	26.73±5.19	28.78±6.30	27.49±5.05	28.72±6.72
HbA1c (%)	5.87±4.10	5.10±0.45	6.89±0.56	4.67±0.58	5.51±1.24	5.23±1.55
FSH (mIU/mL)	16.98±13.62	25.66±16.20	25.93±18.10	17.87±12.55	10.32±7.78	8.26±5.43
LH (IU/mL)	8.63±5.95	12.23±6.82	10.53±7.17	9.17±6.01	6.10±3.84	5.92±3.53
Testosteron (ng/mL)	4.00±3.01	3.61±2.83	4.16±2.73	4.01±3.41	3.96±2.55	4.09±2.14
Microdeletion						
AZFa	13	1	3	5	2	2
AZFb	4	0	1	3	0	0
AZFc	4	0	1	3	0	0
Multiple Deletion	3	1	1	1	0	0
Sperm Retrieval Rate (%)	33.0%	10.0%	2%	12.2%	83.7%	90.7%

**Table 3.** Histopathological and MJ score classification of testicular biopsies with association analyses to sperm retrieval outcomes (n=409).

Johnsen score	Histopathological classification	No. of cases	%	OR of sperm found (95% CI)	*p*-value
8	Normal spermatogenesis	43	10.5	1.00	
7	Hypospermatogensis	82	20.0	0.54 (0.17–1.78)	0.5
6	Spermatogenic Arrest	51	12.5	0.02 (0.01–0.07)	<0.001
5		68	16.6	0.01 (0.00–0.05)	<0.001
4		46	11.2	0.13 (0.00–0.05)	<0.001
3		38	9.3	0.01 (0.00–0.03)	<0.001
2	Sertoli cell only Phenotype	51	12.5	0.01 (0.00–0.02)	<0.001
1	Tubular Fibrosis	30	7.3	0.01 (0.00–0.05)	<0.001

Analysis with Chi-Square and multinomial logistic regression. Odds ratios (OR) and p-values were calculated using multinomial logistic regression, with MJ score 8 (normal spermatogenesis) as the reference group. Lower OR indicates a decreased likelihood of sperm retrieval. Significant if p-value <0.05.

Key variables such as age, BMI, HbA1c, FSH, LH, and testosterone levels were compared across histopathological pattern groups, showed in Table 4 and Figure 2. Although no significant differences were observed in age, BMI, HbA1c, or testosterone levels ( *p*=>0.05 for all), FSH and LH levels varied significantly among groups ( *p*=<0.001). NS and HS exhibited high sperm retrieval rates (SRR), at 93.1% and 88.5%, respectively. In contrast, the rates for SA, SCOP, and TF were significantly lower, with the latter two conditions showing rates as low as 2.1% and 10%, respectively ( *p*=<0.001). The prevalence of microdeletions in the AZF region of the Y chromosome was low across all groups, with no significant ( *p*=0,8) association between specific deletion patterns and histopathological classifications. Patients with TF and SCOP demonstrated the highest median FSH levels (28 and 28.1 mIU/mL, respectively), indicative of more severe spermatogenic dysfunction. Similarly, these groups had significantly ( *p*=<0.001) elevated LH levels compared to other groups. On the other hand, HS showed the closest to normal median values regarding FSH and LH levels of 7.1(2–31) mIU/mL and 5.4 (2–17.4) IU/mL, respectively.

**Table 4.** Relationship between clinical parameters and histopathological patterns.

Total n: 174	TF	SCOP	SA	HS	NS	*p*-value *
N (%)	16 (9.1)	29 (16.6)	80 (45.9)	36 (20.6)	13 (7.4)
Age (years)	34 (30–42)	33 (27–50)	37.5 (26–58)	35.5 (25–61)	37 (27–42)	0.23 ^a^
BMI kg/m ^2^	26.4 (21.25–38.7)	25.5 (19.6–37.2)	27 (19.3–48.3)	26.3 (20–36)	31 (20–44)	0.14 ^a^
SRR (%)	10	2.1	12.8	88.5	93.1	<0.001 ^b^
HbA1c (%)	5.2 (4.2–5.7)	5.3 (4.2–38)	5.2 (4–34)	5.5 (4.1–12.1)	5.5 (4.2–12.5)	0.15 ^a^
Microdeletion *						0.8 ^a^
AZFa	1	2	5	1	2	
AZFb	0	1	3	0	0	
AZFc	0	1	2	0	0	
Multiple deletion	1	1	1	0	0	
FSH (mIU/mL)	28 (0.2–57.3)	28.1 (2.6–67.7)	20.3 (0.3–68.4)	7.1 (2–32)	8.3 (3.6–32.2)	<0.001 ^a^
LH (IU/mL)	10 (0.3–28.4)	11.6 (1.4–43.9)	8.8 (0.1–32.6)	5,4 (2–17.4)	5.6 (1.8–13.8)	<0.001 ^a^
Testosterone (ng/mL)	3.4 (0.3–33.5)	5.6 (0–56.7)	4.3 (0–76.5)	4.4 (0–71)	4.7 (0–63.9)	0.53 ^a^

TF: Tubular Fibrosis; SCOP: Sertoli cell only Phenotype; SA: Spermatogenic Arrest; HS: Hypospermatogenesis; NS: Normal Spermatogenesis; BMI: Body mass index; SRR: Sperm retrieval rates; AZF: Azoospermia factor; FSH: Follicle-stimulating hormone; LH: Luteinizing hormone.

Analysis with:

^a^ ANOVA,

^b^ Analyze with Chi-square,

* Significant if *p*-value <0.05.

## Discussion

The prevalence of male infertility and the associated histological findings in testicular biopsies vary markedly across different regions of the world, influenced by various etiological factors, including social practices, lifestyle, genetic predispositions, and environmental conditions such as infections, chemical exposure, radiation, and heat exposure. ^8, 9^ This study aimed to evaluate histopathological patterns in testicular biopsies of azoospermic men and assess their correlation with clinical and hormonal parameters. The main finding reveals that spermatogenic arrest was the most prevalent histopathological pattern, with significant associations observed between hormonal levels—particularly FSH and LH—and tissue findings.

A testicular biopsy is the primary diagnostic procedure for all testicular-related infertility issues. ^12^ This is not the sole parameter for assessing testicular histopathology patterns; however, it is the most robust predictor of the likelihood of detecting sperm in the testis for therapeutic sperm retrieval in assisted reproductive techniques. ^13^ Moreover, testicular biopsy is crucial in assessing men at risk for carcinoma in situ or testicular cancer, including individuals with idiopathic infertility, a history of cryptorchidism, previous testicular neoplasia, or the presence of concerning clinical or radiological findings such as a nodule or microlithiasis. ^14^ A testicular biopsy may be conducted under local or general anesthesia and can involve either a transcutaneous needle approach or open biopsies from one or multiple sites. Patients with NOA would undergo TESE/micro-TESE under general anesthesia in our practice, depending on the size and previous testicular procedure. During this procedure, the aim is to take samples for sperm retrieval and histopathological examinations at the same time, thus able to move on with ART and evaluate histological changes in the testis if no sperm was found. Due to the variability of lesions between testes and the prevalence of heterogeneous pathological patterns, it is advisable to perform bilateral testicular biopsies in the evaluation of male infertility. ^9, 15– 18^

Various pathological patterns in other international studies are summarized in Table 5. Hypo spermatogenesis is primarily found in our results of 82 cases (20%), similar to other studies before of between 16.7 to 29.4%. ^9, 16, 19– 25^ However, other studies have much smaller findings of 6.6 to 13%. ^17, 20, 26^ and much more significant findings of 41.6 to 55.8%. ^19, 25^ A proportional reduction in the quantities of spermatogonia and primary spermatids characterizes hypo spermatogenesis. In other words, all components of spermatogenesis are present but diminished in quantity. ^15^ Hypo spermatogenesis may clinically correlate with hormonal dysregulation, congenital germ cell deficiency, androgen insensitivity, chemical exposure, and exposure to heat and radiation. ^27^ The variation among studies can be attributed to the differing criteria in patient selection for biopsies. Certain centers reserve testicular biopsies exclusively for patients with azoospermia, whereas others conduct testicular biopsies for patients with either azoospermia or oligospermia. The present study involved testicular biopsy for patients diagnosed with azoospermia.

**Table 5.** Testicular morphologic patterns in other international studies.

Author	No. of subject	Country	Procedure	Year	Histopathological patterns n (%)				
					TF	SCOP	SA	HS	NS
Meinhard et al ^19^	100	UK	TESE	1973	4 (4%)	15 (15%)	46 (46%)	29 (29%)	5 (5%)
Haddad FH et al ^30^	545	Jordan	FNA	1990 – 1995 & 1997 – 2000	155 (28.4)	16 (2.9%)	9 (1.7%)	304 (55.8%)	61 (11.2%)
Abdullah L et al ^16^	100	Saudi Arabia	N/A	2004 – 2010	14 (14%)	16 (16%)	12 (12%)	29 (29%)	13 (13%)
Mushtaq et al ^9^	53	Pakistan	N/A	2011 – 2013	4 (7.54%)	16 (30.18)	8 (15%)	10 (18.86%)	9 (16.78%)
Čamdžić N et al ^17^	219	Bosnia	N/A	2015 – 2023	16 (7.3%)	128 (58.4)	10 (4.6%)	15 (6.8%)	13 (6%)
Allebawi SAH et al ^29^	180	Iraq	N/A	2020 – 2024	18 (10%)	54 (30%)	84 (46.67%)	N/A	24 (13.3%)
Rashed MM et al ^20^	50	Egypt	TESE	2004 – 2006	3 (6%)	17 (34%)	14 (28%)	4 (8%)	12 (24%)
Colgan et al ^21^	142	Canada	TESE	1971 – 1979	N/A	17 (12%)	15 (10,6%)	N/A	29 (20.4%)
Al-Rayess MM et al ^26^	230	Saudi Arabia	N/A	1987 – 1996	12 (5%)	26 (16%)	25 (11%)	30 (13%)	72 (31%)
Siadati S et al ^25^	924	Iran	N/A	1990 – 2013	52 (6.5%)	308 (38.7%)	132 (16.6%)	133 (16.7%)	161 (20.3%)
Brannen GE et al ^23^	48	USA	N/A	1971 – 1977	6 (12.5%)	6 (12.5%)	6 (12.5%)	13 (27%)	17 (35.4%)
Jamal AA et al ^24^	164	Saudi Arabia	N/A	1990 – 2000	N/A	N/A	11 (7%)	41 (25%)	10 (12%)
Seo JT et al ^31^	178	Korea	TESE	1996 – 1999	N/A	80 (44.9%)	24 (13.5%)	74 (41.6%)	N/A
Amin A et al ^22^	34	Iran	FNA	2 years	2 (5.9%)	15 (44.1%)	7 (20.6%)	10 (29.4%)	N/A

TF: Tubular Fibrosis; SCOP: Sertoli cell only Phenotype; SA: Spermatogenic Arrest; HS: Hypospermatogenesis; NS; Normal Spermatogenesis; FNA: Fine needle aspiration; TESE: Testicular sperm extraction; N/A: Not available.

SA is defined as an obstruction in the maturation process of spermatids, resulting in the absence of mature spermatozoa. Affected tubules typically halt at the primary spermatocyte or spermatogonia stage. Clinically, numerous underlying etiologies exist for SA. The arrest may be attributed to genetic factors or external influences. ^20^ Genetic etiologies encompass trisomy, balanced autosomal anomalies (such as translocations and inversions), or deletions on the Y chromosome (Yq11). ^28^ Secondary causes encompass excessive consumption of alcohol or other toxic agents, chronic marijuana use, cytotoxic chemotherapy, and hypogonadotropic hypogonadism. ^15^ The prevalence of SA (accumulation of MJ score 6–3) in this study was 49.5% (203 cases), similar to findings by Meinhard et al. ^19^ in the UK and Allebawi SAH et al. ^29^ in Iraq; these results are much higher than international results of between 1.7 to 28%. ^9, 16, 17, 20– 26, 30, 31^

This study identified 51 cases (12.5%) of SCOP. This finding aligns closely with five additional studies ^16, 19, 21, 23, 26^ that reported analogous figures. Only one study by Haddad FH et al. reported a lower number of SCOP of 2,9%. ^30^ While other studies reported higher results of between 30,18 and 58,4%. ^9, 17, 20, 22, 25, 29, 31^ The disparity in the incidence of SCOP among our study and various international studies remains inadequately elucidated. SCOP should exclusively refer to a consistent pattern in which no germ cells are observed in any profile. Sertoli cell-only Phenotype is an irreversible condition that may be linked to various underlying disorders. These encompass cryptorchidism, orchitis, post-radiation or chemotherapy effects, estrogen or androgen therapy, and chronic hepatopathology as contributing factors. ^15^ Recent studies have implicated structural abnormalities of the Y chromosome, mainly deletions of the human azoospermia factor (AZF) gene on the long arm of chromosome Y, as the primary cause of impaired spermatogenesis and azoospermia. ^28^

Tubular Fibrosis was observed in 30 cases (7,3%). Comparable findings were reported by Meinhard et al., ^19^ Mushtaq et al., ^9^ Čamdžić N et al., ^17^ Allebawi SAH et al., ^29^ Rashed MM et al., ^20^ Siadati S et al., ^25^ and Amin A et al. ^22^ In contrast, higher incidences were documented by Haddad FH et al., ^30^ Abdullah L et al., ^16^ and Brannen GE et al. ^23^ of around (12.5–28.4%). The cause of the discrepancy in the incidence of TF remains unknown; however, variations in biopsy selection criteria among different urologists within the same institution and across various centers contribute to this issue. Another study from Nigeria suggested that prior inflammatory processes, such as previous orchitis, may contribute to the etiology of this condition. ^32^

Conditions such as SCOP and TF exhibit drastically reduced odds for sperm retrieval, with their OR approaching zero. These findings reflect the severity of testicular dysfunction associated with these pathologies. A meta-analysis by Yuen W et al. has found higher SRR in patients with SCOP, as high as 47%. ^33^ The current study showed a significantly lower SRR of 2,1% in patients with SCOP. This difference might be because of the studies included in the meta-analysis, which consist of small sample studies with mainly AZFc microdeletions with SRR rates of 13–100%. ^33^ Intermediate conditions, including SA and HS, demonstrate moderate odds of sperm retrieval, suggesting that partial spermatogenic activity may still provide avenues for successful outcomes in some cases. This result for SA is lower compared to a study by Mehmood S et al. ^34^ (current study: 12.8 vs 32.43%), however the HS pattern is similar (current study: 88.5 vs 89.7%). Several factors might contribute to this difference, such as no further differentiation between early and late MA, variability in testicular Sertoli cell and Leydig cell support, as well as germ cell apoptosis, which can impact the likelihood of successful sperm retrieval, ^35^ and the number and distribution of biopsy samples taken can significantly affect SRR. ^36^ The statistical analysis underscores the significance of histopathological findings in clinical decision-making. The strong association between pathology type and retrieval outcomes provides a framework for predicting patient prognosis and optimizing treatment strategies. This data reaffirms the importance of precise histopathological evaluation as a cornerstone of male infertility management.

The difference in SRR between our study and Ozman et al.’s highlights the significant impact of patient population characteristics and underlying causes of NOA. Our study focused on first-time micro-TESE procedures, which likely included a broader spectrum of NOA cases, encompassing more severe spermatogenic failures, such as SA and SCOP. In contrast, Ozman et al. studied repeat micro-TESE, targeting a population enriched with patients who may have better prognostic features due to prior surgical experience and findings. ^37^ For example, favorable outcomes were observed in Klinefelter’s syndrome (50% SRR) and idiopathic NOA (17.4% SRR) in Ozman et al.’s cohort, suggesting these groups retained residual spermatogenic potential even after a failed initial procedure. However, less favorable etiologies, such as Y microdeletions (20% SRR) and cryptorchidism (18.1% SRR), also contributed to variability in their results. These distinctions emphasize the critical role of the underlying cause of NOA in determining outcomes, as well as the impact of patient selection and procedural history. When comparing SRR between studies, it is essential to consider differences in patient populations, as they reflect biological variability and guide tailored counseling and expectations for success in procedures. A study by Jarvis S et al. showed that testicular fine-needle aspiration (FNA) mapping after failed m-TESE detected sperm in 29.3% of cases, with guided retrieval achieving 100% success, including freezing surplus sperm in 66.7% of patients. This testis-sparing approach identified sperm primarily in peripheral regions, bypassing reliance on visual cues and minimizing extensive surgical procedures like repeated m-TESE. ^37^

FSH and LH levels were notably elevated in patients with TF and SCOP, indicative of significant spermatogenic dysfunction. Median FSH levels for these groups were 28 mIU/mL and 28.1 mIU/mL, respectively, compared to much lower levels in patients with NS or HS. According to a study by Kavoussi PK et al., ^38^ statistically significant higher FSH (more than 22.9±16.6 IU/L) was found in patients with unfavorable (SCOP pattern and early maturation arrest) histopathological patterns compared to a lower FSH level of 13.3±12.0 IU/L in favorable patterns (late maturation arrest and HS). In addition, median LH was also significantly higher in TF and SCOP of (10 IU/mL and 11,6 IU/mL), respectively, as compared to SA, HS, and NS. This suggests a disruption in the feedback mechanisms between the testis and the hypothalamus-pituitary axis, negatively correlated with spermatogenic function. ^38^ Elevated LH levels are further associated with Leydig cell hyperplasia and reduced testosterone levels, reinforcing these endocrine markers’ diagnostic and prognostic value. ^39^ These hormones are valuable biomarkers in diagnosing and predicting the severity of male infertility based on testicular histopathology. Nonetheless, testosterone may still offer supportive diagnostic value when interpreted alongside gonadotropins. When interpreted collectively, these hormonal markers can serve as valuable non-invasive indicators of spermatogenic status. An FSH threshold above 22 mIU/mL may reliably predict unfavorable histological outcomes, offering potential utility as a screening tool for identifying candidates with a low likelihood of successful sperm retrieval. ^38^

This study underscores the clinical relevance of integrating histopathological and hormonal findings in the management of azoospermic men. Testicular biopsy, combined with FSH and LH profiling, provides a robust framework for counseling patients regarding their realistic prospects with assisted reproductive technologies (ART). ^39^ Identifying severe histological patterns such as Sertoli cell-only phenotype or tubular fibrosis—especially when accompanied by elevated FSH—can help avoid unnecessary repeat interventions. By incorporating hormonal thresholds into pre-biopsy screening, clinicians may more effectively stratify candidates for micro-TESE, improving timing and patient selection while potentially reducing the need for invasive procedures in those with minimal retrieval potential. ^38, 40^ These findings have practical implications for clinical protocols in resource-limited settings, where access to advanced reproductive technologies may be limited. By integrating hormonal profiling—particularly FSH and LH levels—with histopathological assessment, clinicians can more effectively triage patients for invasive procedures like micro-TESE. Establishing hormone-based thresholds as non-invasive predictors of unfavorable pathology could reduce unnecessary surgeries, optimize resource allocation, and support more personalized counseling, ultimately improving care quality and cost-effectiveness in low-resource healthcare environments. ^40^

This study possesses several notable strengths, making it a valuable contribution to the field of male infertility research. A large sample size of 409 testicular biopsies provides robust statistical power and reliable findings. Additionally, its emphasis on bilateral testicular biopsies improves diagnostic accuracy by addressing histopathological discrepancies between testes. By situating its findings within data from Indonesia and international studies, the research enhances understanding of global histopathological variations while addressing the Indonesian population’s unique demographic and healthcare challenges. These strengths underscore the study’s clinical relevance in guiding patient counseling, treatment planning, and improving outcomes in male infertility management. On the other hand, there are some limitations regarding this study. Firstly, the study relies on retrospective data, which may introduce bias and limit the ability to establish causality. Secondly, we only encounter a few patients with Y-chromosome microdeletions; therefore, we cannot evaluate this population further. These abnormalities might significantly affect the hormonal profile ^40^ and SRR ^41^ in azoospermic patients. While this is the largest study in Indonesia, data from 2 hospital might not be a true representative of the entire population. The retrospective nature also introduced specific biases although this is not a major issue in this correlational study. x Future research should consider prospective, multicenter studies to improve population representation and allow for controlled evaluation of causative factors. Genetic screening, including Y-chromosome microdeletion analysis, should be incorporated to better understand its impact on histopathological patterns and hormonal profiles.

## Conclusion

The study on testicular biopsies in azoospermic men within an Indonesian cohort highlights the intricate relationship between histopathological findings and fertility potential. Elevated FSH and LH levels were strongly associated with severe spermatogenic dysfunction, particularly in SCOP and TF cases. The study underscores the importance of precise histopathological evaluations in optimizing fertility treatment outcomes and provides valuable insights into Indonesia’s unique demographic and healthcare context.

## Data Availability

Data supporting the findings of this study are available upon reasonable request from the corresponding author (Ponco Birowo, email: ponco.birowo@gmail.com) due to ethical restrictions. The data include de-identified clinical, laboratory, and histopathological parameters of patients with non-obstructive azoospermia. Access to the data will be granted following institutional data protection regulations and upon obtaining necessary ethical approvals. No publicly available extended data are currently associated with this study. Supplementary materials such as the histopathological scoring table, blank consent form, or study protocols can be provided upon reasonable request to the corresponding author (Ponco Birowo, email: ponco.birowo@gmail.com). Data sharing is subject to institutional data protection policies and requires prior ethical approval.
