# Supplementary material for: Uncovering fertility potential: Clinical and histopathological insights from testicular biopsies in azoospermic men in a large Indonesian cohort
Source: F1000Res. 2026 Mar 18;15:25. Originally published 2026 Jan 7. [Version 2] doi: 10.12688/f1000research.166812.2 (PMC13182704; doi:10.12688/f1000research.166812.2)
Supplement: Peer review history

# Reviewer report: Reviewer response for version 2

**DOI:** 10.5256/f1000research.197124.r468660

**Published:** 2026-03-19

**Authors:** Hideyuki Kobayashi

**Affiliations:** 1 Department of Urology, Toho University faculty of Medicine, Tokyo, Japan

I have reviewed the revised version of the manuscript and the authors’ responses to the reviewers’ comments. In my opinion, the authors have addressed the major concerns appropriately. The revisions have improved the histopathological classification, clarified the methods, and strengthened the clinical interpretation through additional ROC and multivariate analyses. The remaining limitations are appropriately acknowledged.

Is the work clearly and accurately presented and does it cite the current literature?

Yes

If applicable, is the statistical analysis and its interpretation appropriate?

Yes

Are all the source data underlying the results available to ensure full reproducibility?

Yes

Is the study design appropriate and is the work technically sound?

Yes

Are the conclusions drawn adequately supported by the results?

Yes

Are sufficient details of methods and analysis provided to allow replication by others?

Yes

Reviewer Expertise:

Male infertility

I confirm that I have read this submission and believe that I have an appropriate level of expertise to confirm that it is of an acceptable scientific standard.

# Reviewer report: Reviewer response for version 1

**DOI:** 10.5256/f1000research.183856.r457414

**Published:** 2026-02-16

**Authors:** Hideyuki Kobayashi

**Affiliations:** 1 Department of Urology, Toho University faculty of Medicine, Tokyo, Japan

This manuscript presents a large retrospective cohort study evaluating histopathological patterns and hormonal profiles in azoospermic men undergoing bilateral testicular biopsy in Indonesia. The dataset of 409 cases represents an important regional contribution, and the emphasis on bilateral biopsy and sperm retrieval rate (SRR) adds clinical relevance. However, several methodological and interpretative issues should be addressed before the manuscript can be considered for approval.

 Major comments

1. High proportion of spermatogenic arrest (SA)

The reported prevalence of spermatogenic arrest (approximately 49–50%) appears substantially higher than that described in most previously published cohorts, where SA generally ranges between 10% and 30%. In the present study, Modified Johnsen (MJ) scores 6–3 are collectively categorized as SA. This broad classification likely encompasses both early and late maturation arrest, which are biologically and prognostically distinct entities.

 Subclassification of SA into early (arrest at primary spermatocyte level) and late (arrest at round spermatid stage) maturation arrest is strongly recommended. These subtypes are known to have different sperm retrieval rates and may explain the relatively low overall SRR (approximately 12%) observed in this SA group. Without this distinction, comparison with international literature becomes challenging and may artificially inflate the SA proportion.

 The authors should clarify:

- Whether early and late maturation arrest were differentiated histologically.
- How many tubules were evaluated per biopsy.
- Whether interobserver variability was assessed.

1. Lack of predictive modeling (ROC / Cut-off Analysis)

While significant associations between FSH/LH levels and histopathological patterns are reported (p < 0.001), the manuscript remains largely correlational. For clinical applicability, it would be highly valuable to provide receiver operating characteristic (ROC) curve analysis to determine clinically meaningful cut-off values for predicting unfavorable histopathology or failed sperm retrieval.

 For example:

- What FSH threshold predicts SCOP/TF with acceptable sensitivity and specificity?
- Can LH independently predict severe pathology?
- What is the area under the curve (AUC) for these parameters?

Including such analyses would substantially strengthen the translational value of the study.

1. Multivariate analysis for sperm retrieval

The manuscript reports multinomial logistic regression for histopathological classification; however, it is unclear whether hormonal parameters independently predict sperm retrieval after adjusting for histopathology.

 A multivariate logistic regression model evaluating independent predictors of SRR (including age, BMI, FSH, LH, testosterone, and histopathology) would significantly enhance the robustness of the findings. Currently, it is difficult to determine whether hormonal markers provide additional predictive value beyond biopsy results.

1. Bilateral discordance analysis

The authors report a discordant histopathological pattern in 18.5% of cases. This is an important finding, yet its clinical implications are not sufficiently explored.

 Specifically:

- What were the SRRs in discordant versus concordant cases?
- In discordant cases, was sperm retrieved preferentially from the more favorable side?
- How was discordance handled in statistical modeling?

Further elaboration on these points would reinforce the argument supporting bilateral biopsy.

1. Minor but important issues

- The mean age is reported as “366.7 years” in the Results section, which appears to be a typographical error.
- The claim of being the “largest dataset” in Indonesia should be cautiously phrased, as data were derived from two centers.
- HbA1c is included in the analysis but not meaningfully discussed; its relevance should be clarified or the variable reconsidered.

Is the work clearly and accurately presented and does it cite the current literature?

Yes

If applicable, is the statistical analysis and its interpretation appropriate?

Yes

Are all the source data underlying the results available to ensure full reproducibility?

Yes

Is the study design appropriate and is the work technically sound?

Yes

Are the conclusions drawn adequately supported by the results?

Yes

Are sufficient details of methods and analysis provided to allow replication by others?

Yes

Reviewer Expertise:

Male infertility

I confirm that I have read this submission and believe that I have an appropriate level of expertise to confirm that it is of an acceptable scientific standard, however I have significant reservations, as outlined above.

# Response

**Published:** 2026-02-24

**Authors:** Ponco Birowo

**Affiliations:** Department of Urology Faculty of Medicine Universitas Indonesia/ CIpto Mangunkusumo Hospital, Indonesia

First of all, I thank you for reviewing our study. I would like to respond to the revisions noted by the reviewer:

 1. High proportion of spermatogenic arrest (SA) and subclassification into early/late maturation arrest:

 We thank the reviewer for this critical observation. We agree that grouping all SA cases (MJ 3–6) inflated our overall prevalence compared to international cohorts. Due to the retrospective nature of the historical pathology reports spanning 14 years, SA was initially categorized broadly without formal subclassification. However, to address this, we have now stratified the specific MJ scores in the Discussion section to differentiate the arrest stages. We clarified that only 12.5% of cases (MJ 6) represented late maturation arrest, while 27.8% (MJ 4 and 5) represented early arrest, and 9.3% (MJ 3) showed pre-meiotic arrest. We have also updated the Methods to clarify that, typically, 20 to 50 tubules are evaluated per biopsy. As we could not retrospectively calculate Cohen's Kappa for interobserver variability, we have explicitly stated this as a limitation.

 2. Lack of predictive modeling (ROC / Cut-off Analysis):

 We greatly appreciate this suggestion to enhance the translational value of the study. We have now performed ROC curve analyses to determine the predictive value of FSH and LH for severe histopathology (SCOP and TF). For predicting SCOP, FSH demonstrated an AUC of 0.693 (p<0.001), with a clinical cut-off of 14.75 mIU/mL providing 78.4% sensitivity. For predicting Tubular Fibrosis, FSH demonstrated an AUC of 0.672 (p=0.003) with an optimal cut-off of 14.95 mIU/mL. These clinically meaningful cut-offs, along with their respective sensitivities and specificities, have been added to the Results and Discussion sections, and the ROC curves have been included as Figures 3 and 4.

 3. Multivariate analysis for sperm retrieval:

 We thank the reviewer for this sugestion. We have conducted a multivariate logistic regression model (now included as Table 6) evaluating independent predictors of successful sperm retrieval, incorporating age, BMI, FSH, LH, testosterone, and histopathological classification. The analysis revealed that hormonal parameters, including FSH (p=0.747) and LH (p=0.074), were not independent predictors of sperm retrieval after adjusting for histopathology. The histopathological patterns (TF, SCOP, and SA) remained the sole independent predictors of retrieval failure (p<0.001 for all). We have updated the Results and Discussion to emphasize that endocrine profiling cannot replace the diagnostic accuracy of a testicular biopsy.

 4. Bilateral discordance analysis:

 We agree that the 18.5% discordance rate is an important finding that supports bilateral biopsies. As noted in the Methods, in cases of discordance, the higher MJ score (the more favorable side) was used for statistical classification. Unfortunately, due to the extended retrospective nature of the surgical records, the specific laterality of successful sperm extraction was not consistently documented in the operative notes for these discordant cases. Consequently, we could not reliably calculate side-specific SRRs. We have added a sentence to the limitations section acknowledging this constraint.

 5. The mean age is reported as “366.7 years”:

 We apologize for this typographical error. It has been corrected to "36 ± 6.7 years" in the Results section.

 6. The claim of being the “largest dataset” in Indonesia should be cautiously phrased.

 We completely agree. We have revised the phrasing throughout the manuscript (including the Abstract and Limitations) to state that this is "one of the largest datasets" or a "substantial cohort," reflecting that the data was derived from two centers and may not represent the entire population.

 7. HbA1c is included in the analysis but not meaningfully discussed.

 We appreciate the reviewer highlighting this. We have expanded the Discussion section to explicitly contextualize the HbA1c findings. We introduced the concept of the "metabolic paradox" in surgical sperm retrieval, explaining that while systemic HbA1c strongly impairs *ejaculated* sperm quality, it frequently fails to predict micro-TESE success because surgical retrieval bypasses the hostile post-testicular environment. We clarified that while our p-value was not significant (p=0.15), the data suggests HbA1c should not serve as a prognostic gatekeeper to deny surgical fertility interventions.

# Reviewer report: Reviewer response for version 1

**DOI:** 10.5256/f1000research.183856.r450727

**Published:** 2026-02-06

**Authors:** Doddy Moesbadianto Soebadi

**Affiliations:** 1 Department of Urology, Faculty of Medicine, Universitas Airlangga, Surabaya, East Java, Indonesia

This study is an important source for the literature and as a largest dataset concerning azoospermia in Indonesia. Also it provides a comprehensive histopathological and hormonal profile of azoospermic patients in the country, while also containing the diagnostic value of bilateral testicular biopsies – an area often underexplored, especially in Asia countries. The histopathological and hormonal data as well as the data of microdeletions and sperm retrieval rates make this study more valuable.

 I recommend to approve this paper as an important addition of the world literature.

Is the work clearly and accurately presented and does it cite the current literature?

Yes

If applicable, is the statistical analysis and its interpretation appropriate?

Yes

Are all the source data underlying the results available to ensure full reproducibility?

Yes

Is the study design appropriate and is the work technically sound?

Yes

Are the conclusions drawn adequately supported by the results?

Yes

Are sufficient details of methods and analysis provided to allow replication by others?

Yes

Reviewer Expertise:

Clinical and basic research in Andrology.

I confirm that I have read this submission and believe that I have an appropriate level of expertise to confirm that it is of an acceptable scientific standard.
